# M-Finder: Uncovering functionally associated proteins from interactome data integrated with GO annotations

**DOI:** 10.1186/1477-5956-11-S1-S3

**Published:** 2013-11-07

**Authors:** Young-Rae Cho, Marco Mina, Yanxin Lu, Nayoung Kwon, Pietro H Guzzi

**Affiliations:** 1 Bioinformatics Program, Department of Computer Science, Baylor University, Waco, TX, USA; 2 Department of Information Engineering, University of Padova, Padova, Italy; 3 MPBA, Fondazione Bruno Kessler, Trento, Italy; 4 Department of Computer Science, Rice University, Houston, TX, USA; 5 College of Pharmacy, University of Iowa, Iowa City, IA, USA; 6 Department of Surgical and Medical Sciences, University of Catanzaro, Catanzaro, Italy

## Abstract

**Background:**

Protein-protein interactions (PPIs) play a key role in understanding the mechanisms of cellular processes. The availability of interactome data has catalyzed the development of computational approaches to elucidate functional behaviors of proteins on a system level. Gene Ontology (GO) and its annotations are a significant resource for functional characterization of proteins. Because of wide coverage, GO data have often been adopted as a benchmark for protein function prediction on the genomic scale.

**Results:**

We propose a computational approach, called M-Finder, for functional association pattern mining. This method employs semantic analytics to integrate the genome-wide PPIs with GO data. We also introduce an interactive web application tool that visualizes a functional association network linked to a protein specified by a user. The proposed approach comprises two major components. First, the PPIs that have been generated by high-throughput methods are weighted in terms of their functional consistency using GO and its annotations. We assess two advanced semantic similarity metrics which quantify the functional association level of each interacting protein pair. We demonstrate that these measures outperform the other existing methods by evaluating their agreement to other biological features, such as sequence similarity, the presence of common Pfam domains, and core PPIs. Second, the information flow-based algorithm is employed to discover a set of proteins functionally associated with the protein in a query and their links efficiently. This algorithm reconstructs a functional association network of the query protein. The output network size can be flexibly determined by parameters.

**Conclusions:**

M-Finder provides a useful framework to investigate functional association patterns with any protein. This software will also allow users to perform further systematic analysis of a set of proteins for any specific function. It is available online at http://bionet.ecs.baylor.edu/mfinder

## Background

PPI data have a central role in understanding functional behavior of proteins. Recent high-throughput techniques [1,2] have generated the interactome, an entire set of PPIs on the genomic scale. The accumulative PPI data sets of several model organisms are publicly available in a number of open databases [3]. Availability of interactomes has introduced a new paradigm towards functional characterization of proteins [4,5]. However, the high-throughput experimental and computational methods have also made their outcomes less reliable, causing the presence of a large fraction of false-positive interactions. Therefore, curation of current PPI data sets by integration of other data sources has been strongly demanded [6,7].

In the last decade, a wide range of computational algorithms have been introduced to predict protein complexes or functional modules from genome-wide PPI networks [8-12]. A functional module represents a set of proteins which participate in the same biological processes. Because of unreliability of the PPI data set, integrative approaches have also been applied to uncover functional organizations and their structures which are hidden in the PPI networks [13]. As one of the integrative approaches, the functional association level (or functional consistency) between interacting proteins can be quantified by a semantic similarity measure which represents a model for measuring closeness in meaning between two or more ontological terms. The semantic measures may be extended to proteins by using the terms to which they are annotated [14]. The use of GO and its annotations [15] has been commonly suggested to compute the semantic similarity of each interacting protein pair. Higher semantic similarity between two sets of GO terms of two interacting proteins, respectively, indicates that the proteins are more closely associated with each other in terms of their functions. Although there exist some unreliable sources on GO annotation data (e.g., the results from high-throughput experimental and computational methods), they are often adopted as a benchmark for functional characterization of proteins because of their wide coverage on the genomic scale over various model organisms.

A previous study [16] proposed a computational approach for functional association pattern mining. The proposed method employs a two-step strategy. First, up-to-date PPI data sets are extracted from the BioGRID database [17] and weighted by two advanced semantic similarity metrics, called simICNP and simICND. In this article, we evaluate their performance by comparing to other previous methods. We investigate whether each interacting pair agrees on other biological features, such as sequence similarity, the presence of common Pfam domains, and core PPIs.

Second, the core of functional association pattern mining is an information flow-based algorithm that runs on the weighted genome-wide PPI network. When a protein is given by a user, this algorithm generates a group of proteins functionally associated with the protein and their functional links by random walk simulation. Since this algorithm has the advantage of being remarkably efficient, it is well-applicable to web-based tools. In this article, we introduce web application software, called M-Finder, that reconstructs a functional association network from a protein specified by a user. This interactive web-based tool takes a protein entered by a user in a query (using systematic names or gene symbols as protein identifiers) and visualizes a network generated by dynamic information propagation starting from the query protein. The visualized network represents a functional linkage pattern associated with the protein of interest. The output network size can be flexibly determined by the parameters that a user specifies. Moreover, M-Finder provides detailed ontological and experimental information of each interactor and each interaction, which can be obtained through the hyperlinks on the visualized networks. M-Finder will allow users to characterize functional mechanisms of proteins on the genomic scale in a systematic perspective.

## Methods

### Semantic similarity

#### Survey of semantic similarity methods

An ontology provides well-defined, structured and computable semantics of domain knowledge [18]. Because of the need for consistent description related to genes and gene products across species, GO has been launched by a collaborative effort to build complete ontologies in the biological domain [19]. Semantic similarity is a function to measure closeness in meaning between ontological terms [14]. Over the past few years, various methods to compute semantic similarity using GO and its annotation data have been proposed [20-22]. The semantic similarity scores have been applied to quantify functional similarity between proteins. According to the components used in GO, we can group the existing methods into four broad categories: edge-based methods (measuring path length between two terms), node-based methods (counting common ancestor terms between two terms), annotation-based methods (measuring information contents of two terms), and integrative methods.

Suppose we measure the semantic similarity between two GO terms *t*_1 _and *t*_2 _having the annotation of two proteins of interest, respectively. First, edge-based methods explore the paths between GO terms in a DAG (Directed Acyclic Graph) structure of GO. For instance, we can compute the shortest path length between *t*_1 _and *t*_2_. Since each ontology has a different scale, the shortest path length between two terms can be normalized by the ontology depth, i.e., the greatest length among the shortest paths from the root to leaf terms. Another example in this category is to measure the depth to the most specific common ancestor term (called SCA) of *t*_1 _and *t*_2_. The greater depth to SCA indicates higher semantic similarity between *t*_1 _and *t*_2_. This method can be normalized by the average depth to the individual GO terms, *t*_1 _and *t*_2_. However, it has been observed that these methods are not appropriate for assessing functional similarity of proteins because GO has inherent complex relationships among GO terms and it cannot be guaranteed that the edges in GO represent the same quantity of specificity.

Second, node-based methods measure the overlap between two sets of ancestor terms of *t*_1 _and *t*_2_, respectively [23]. The greater intersection of the two sets, the higher semantic similarity between *t*_1 _and *t*_2_. This approach can be normalized by the union of two ancestor term sets of *t*_1 _and *t*_2_, called simUI [24].

$$sim_{simUI}\left(t_{1},t_{2}\right)=\frac{C\left(t_{1}\right)\cap C\left(t_{2}\right)}{C\left(t_{1}\right)\cup C\left(t_{2}\right)},$$

where *C*(*t*_1_) is a set of all ancestor terms of *t*_1_.

Third, annotation-based methods utilize the number of annotating proteins on each GO term to infer the term specificity. In Information Theory, information content of a concept *c *is defined as the negative log likelihood of *c*. In the same manner, information content of a GO term *t_i _*can be described as - log *P *(*t_i_*), where *P *(*t_i_*) is the proportion of annotating proteins to *t_i_*. In order to make information contents of any GO terms represent their specificity, if a protein is annotated to *t_i_*, we should annotate the protein to all ancestor terms of *t_i _*on the paths towards the root. Then, the smaller number of annotating proteins a GO term has (i.e., the greater information content it has), the more specific it is. The root term of GO is the least specific because it has the maximum number of annotating proteins. Resnik's method [25] computes the semantic similarity between *t*_1 _and *t*_2 _by the greatest information content of common ancestor terms of *t*_1 _and *t*_2_. In other words, this method estimates the specificity of SCA.

$$sim_{Resnik}\left(t_{1},t_{2}\right)=\overset{}{\underset{t_{0}\in C\left(t_{1},t_{2}\right)}{\text{max}}}\left(-\text{log}\;P\left(t_{0}\right)\right),$$

where *C*(*t*_1_, *t*_2_) is a set of all common ancestor terms of *t*_1 _and *t*_2_. Lin [26] proposed a normalized formula of Resnik's method by the average information content of *t*_1 _and *t*_2_.

$$sim_{Lin}\left(t_{1},t_{2}\right)=\underset{t_{0}\in C\left(t_{1},t_{2}\right)}{\text{max}}\left(\frac{2\times \text{log}P\left(t_{0}\right)}{\text{log}\;P\left(t_{1}\right)+\text{log}\;P\left(t_{2}\right)}\right).$$

Jiang and Conrath [27] calculate differences of the information contents between SCA and the individual GO terms, *t*_1 _and *t*_2_, and measure the semantic similarity between *t*_1 _and *t*_2 _by the inverse of the differences. The smaller the differences of the information contents between SCA and *t*_1 _and between SCA and *t*_2_, the more similar *t*_1 _and *t*_2_.

$$sim_{Jiang}\left(t_{1},t_{2}\right)=\frac{1}{{\text{min}}_{t_{0}\in C\left(t_{1},t_{2}\right)}\left(2\times \text{log}\;P\left(t_{0}\right)-\text{log}\;P\left(t_{1}\right)-\text{log}\;P\left(t_{2}\right)\right)+1}.$$

Schlicker et al. [28] proposed a combined method of Resnik's and Lin's methods, which is called simRel. If SCA is defined as the term where two paths towards the root from *t*_1 _and *t*_2 _converge, multiple SCAs of *t*_1 _and *t*_2 _generally occur in a DAG structure since each GO term has multiple parent terms. Couto et al. [29] defined a set of all SCAs of pairwise paths towards the root from *t*_1 _and *t*_2 _as common disjunctive ancestors. They proposed add-on semantic similarity methods, GraSM which averages the information contents of common disjunctive ancestor terms and DiShln which is a slight modification of GraSM [30].

Finally, many integrative approaches of two different categories have recently been proposed to achieve higher accuracy in measuring functional similarity of proteins. Wang et al. [31] introduced a combination of the normalized node-based method and the edge-based method. Their semantic similarity measure, called G-SESAME, scores a protein pair by the common GO terms having the annotations of the proteins, but gives different weights to the common GO terms according to their depth. Pesquita et al. [32] proposed simGIC which integrates the normalized node-based method with information contents. Instead of counting the common terms, simGIC sums the information contents of the common terms.

$$sim_{simGIC}\left(t_{1},t_{2}\right)=\frac{\sum_{t_{i}\in \;\;C\left(t_{1}\right)\cap \;\;C\left(t_{2}\right)}\text{log}\;P\left(t_{i}\right)}{\sum_{t_{j}\;\in \;\;C\left(t_{1}\right)\;\cup \;\;C\left(t_{2}\right)}\text{log}\;P\left(t_{j}\right)},$$

where *C*(*t*_1_) is a set of all ancestor terms of *t*_1_. IntelliGO [33] integrates the edge-based method with information contents as weight. Jain and Bader [34] proposed an integrative approach, called TCSS, which integrates a clustering technique with a semantic similarity measure. Clustering of GO terms yields a set of subgraphs of GO. Semantic similarity is weighted to allow for inclusion of two terms in the same subgraph.

Previous experiments [32,34] have shown that Resnik's method (an annotation-based method), simUI (a node-based method), and simGIC as a combination of Resnik's and simUI have relatively good performance. We listed all methods in the four categories in Table 1.

**Table 1:** Summary of semantic similarity methods in four categories.

Method	Description
**Edge-based**	
Path-length	Path-length between two GO terms
Depth	Depth to SCA divided by average depth to two GO terms

**Node-based**	
TO	The number of common ancestors of two GO terms
simUI	Common ancestors divided by union of ancestor sets of two GO terms

**Annotation-based**	
Resnik	IC of SCA of two GO terms
Lin	IC of SCA divided by average IC of two GO terms
Jiang	Sum of differences of ICs between SCA and two GO terms
GraSM	Average IC of all disjunctive common ancestors of two GO terms
simRel	Combination of Resnik's and Lin's methods
simICND	Combination of Resnik's and Jiang's methods

**Integrative**	
G-SESAME	Combination of common ancestor terms and their depth
simGIC	Combination of simUI and ICs of ancestor terms
IntelliGO	Combination of depth to two GO terms and ICs of ancestor terms
TCSS	Combination of Resnik's method and a clustering technique
simICNP	Combination of Resnik's method and path-length between two GO terms

We group the existing semantic similarity methods and two proposed measures (simICND and simICNP) into four broad categories according to the components used in GO. SCA denotes the most specific common ancestor term of two GO terms that have the annotation of two proteins of interest, respectively. IC denotes the information content of a GO term.

Our aim is to quantify functional similarity between two proteins using semantic similarity scores of GO terms. Since each protein is typically annotated to multiple GO terms, a single semantic similarity score between two proteins, *p*_1 _and *p*_2_, should be derived from multiple semantic similarity scores between two sets of GO terms, *S*_1 _and *S*_2_. Three different ways are commonly used for aggregating semantic similarity scores between pairwise combinations of the GO terms having the annotation of *p*_1 _and *p*_2_: averaging, maximum, and the best-match averaging (BMA). The averaging method is to compute the average semantic similarity score of all possible pairwise combinations of the terms in *S*_1 _and *S*_2_.

$$sim_{avg}\left(p_{1},p_{2}\right)=\frac{\underset{t_{1}\in S_{1},t_{2}\in S_{2}}{\sum }sim\left(t_{1},t_{2}\right)}{\left|S_{1}|\times |S_{2}\right|}.$$

The maximum method is to select the maximal semantic similarity score out of them.

$$sim_{max}\left(p_{1},p_{2}\right)=\underset{t_{1}\in S_{1,}t_{2}\in S_{2}}{\text{max}}sim\left(t_{1},t_{2}\right).$$

Finally, we can compute the average of all pairwise best-matches [35] such as:

(1) $$sim_{BMA}\left(p_{1},p_{2}\right)=\frac{\sum_{t_{1}\in S_{1}}{\text{max}}_{t_{2}\in S_{2}}sim\left(t_{1},\overset{}{\underset{2}{t}}\right)+\sum_{t_{2}\in S_{2}}{\text{max}}_{t_{1}\in S_{1}}sim\left(t_{1},t_{2}\right)}{\left|S_{1}|+|S_{2}\right|}.$$

Previous studies have also observed that the BMA approach is the best for estimating functional similarity between two proteins which perform multiple functions.

#### Improvement of semantic similarity

Additional improvements of semantic similarity measurement by integrating two orthogonal features have been proposed. Resnik's method focuses on the commonality of two GO terms *t*_1 _and *t*_2_, not a difference between them. In contrast, Lin's and Jiang's methods measure their difference only. In particular, Lin's model reflects a significant bias towards higher scores when the set of annotating proteins on SCA is similar to those on *t*_1 _and *t*_2_. This case commonly occurs in GO because of the shallow annotation problem [22]. To enhance the performance of these annotation-based methods, Resnik's semantic similarity of *t*_1 _and *t*_2 _can be normalized by their distance. Two integrative approaches, simICNP and simICND, presented in [16], use the distance between *t*_1 _and *t*_2 _as the normalization factor.

(2) $$sim_{ICNP}\left(t_{1},t_{2}\right)=\frac{-\text{log}\;P\left(t_{0}\right)}{len\left(t_{1},t_{2}\right)+1},$$

and

(3) $$sim_{ICND}\left(t_{1},t_{2}\right)=\frac{-\text{log}\;P\left(t_{0}\right)}{2\cdot \text{log}\;P\left(t_{0}\right)-\text{log}\;P\left(t_{1}\right)-\text{log}\;P\left(t_{2}\right)+1},$$

where *t*_0 _is SCA of *t*_1 _and *t*_2 _which has the greatest information content among their common ancestor terms. The normalization factor of simICNP is the path length between *t*_1 _and *t*_2 _in the ontology, whereas that of simICND is the difference of information contents of *t*_1 _and *t*_2_. simICNP works better when the ontology has precise information of relationships between specific terms. In contrast, simICND has a better performance when specific terms in the ontology have a sufficient amount of annotations. Therefore, simICND and other annotation-based semantic similarities such as Resnik's method and Lin's method have high accuracy of measuring functional consistency between two proteins for well-studied model organisms. However, their weakness is low accuracy for rarely-studied organisms.

In this study, we make a complete evaluation of simICNP, simICND and other competing semantic similarity methods in a biological perspective, as shown in the next section. For both simICNP and simICND, we use the BMA approach in Formula 1 to achieve functional similarity scores of all interacting protein pairs.

### Discovering functional associations

To discover the functional associations (or functional linkage) of a given protein, we apply the information flow algorithm, presented in [36], to the weighted PPI network. The algorithm is based on the path strength model defined as the product of edge weights divided by node degrees on the path. This model describes that a path, i.e. a series of proteins directly connected, generally has high strength with high edge weights and low node degrees on the path. Starting from a protein that a user specifies, information flow traverses a genome-wide PPI network through all links and updates repeatedly the functional influence score on each protein using the path strength model. The major strength of this approach is high efficiency in scoring functional influence of the user-specified protein on any other proteins in a PPI network with complex connectivity. Recursive random walk computation in this algorithm runs extremely faster than enumerating all possible paths from the user-specified protein to other proteins.

This approach allows to set a parameter to terminate the information flow on a path. As information flows continuously through the links, the algorithm generates monotonically decreasing functional influence scores according to the path strength model. When the score is lower than a user-specified threshold, the flow stops on the specific path. The algorithm finally terminates when any link does not have a flow. The lower the threshold, the longer the algorithm runs.

This approach requires additional parameter to return a functional association network which represents a subgraph of the genome-wide PPI network. When the information flow terminates, the algorithm collects the proteins and their links whose functional influence scores are greater than a user-specified threshold. This threshold thus determines the size of the generated network. As the threshold decreases, the algorithm returns a larger functional association network.

## Results and discussion

### Assessment of semantic similarity

#### CESSM test

Following the underlying idea that functionally related proteins present common physical attributes, semantic similarity measures are usually assessed by evaluating their agreement to other biological features, such as sequence similarity, the presence of common domains, and protein-protein interactions [22]. We employed CESSM to compare the proposed semantic similarity measures, simICND and simICNP, to other state-of-the-art methods with respect to their performance of scoring functional similarity between proteins.

CESSM [37] is a ready-to-use online tool that evaluates the relationship between semantic measures and other similarities based on sequence, Pfam family [38], and EC (Enzyme Commission) classification [39] on a predefined set of 13,430 protein pairs of *S. cerevisiae*. The selected protein pairs are annotated not only to GO terms but also in Pfam and KEGG databases. For each feature, a quantitative similarity score is calculated for each protein pair. The Pearson correlation is then used to evaluate the agreement between semantic similarity and the other features on the whole data set. The higher the correlation, the better the tested measure. It has been pointed out that, in general, the relationship between sequence and semantic similarity is not linear, and therefore Pearson correlation might not be the best measure to assess their agreement. Indeed, in the comparison with sequence similarity, CESSM also considers resolution as a quality measure [32]. Intuitively, the resolution measures the intensity with which variations in sequence similarity have effects on semantic similarity. A measure with a higher resolution is likely to yield a greater variation, in terms of semantic similarity, between protein pairs with low and high sequence similarity.

To score the semantic similarities of selected protein pairs, we used two ontologies in GO, biological process (BP) and molecular function (MF), separately. GO annotation data have been collected by published results from various high-throughput approaches including both experimental and computational analysis. GO provides evidence codes to indicate the types of methods that create the annotation. All evidence codes have been curated manually with the exception of Inferred from Electronic Annotation (IEA). We thus tested two different sets of semantic similarity scores measured with and without IEA annotations, respectively.

Table 2, 3, 4, 5 show the CESSM test results of eight different semantic similarity methods. They include six previous methods: Resnik's, Lin's and Jiang's methods, simUI, simGIC and G-SESAME. The first three methods are in the annotation-based group, simUI is a node-based method, and the last two are integrative approaches. Previous studies [21,32,34] have observed that these methods have relatively good performance in terms of assessing functional similarity between proteins. The tables also incorporate the results of two proposed methods, simICNP and simICND.

**Table 2:** CESSM results of semantic similarities in BP ontology with IEA annotations.

Similarity	Resnik	Lin	Jiang	simUI	simGIC	G-SESAME	simICNP	simICND
Sequence	**0.740**	0.637	0.586	0.730	**0.773**	0.684	0.719	0.710
Pfam	0.459	0.373	0.332	0.451	0.455	0.482	**0.506**	**0.509**
ECC	0.444	0.435	0.371	0.402	0.398	0.430	**0.458**	**0.472**
Resolution	0.900	0.933	0.335	0.863	0.837	0.945	**0.973**	**0.992**

The semantic similarities in BP ontology from eight different methods were compared in terms of correlations with the similarities of other functional features. To measure semantic similarity, IEA annotations were included.

**Table 3:** CESSM results of semantic similarities in BP ontology without IEA annotations.

Similarity	Resnik	Lin	Jiang	simUI	simGIC	G-SESAME	simICNP	simICND
Sequence	**0.727**	0.627	0.533	0.695	**0.736**	0.618	0.696	0.695
Pfam	0.451	0.381	0.274	0.425	0.438	0.371	**0.452**	**0.453**
ECC	**0.426**	0.422	0.411	0.382	0.389	0.377	0.417	**0.424**
Resolution	0.893	0.912	0.357	0.883	0.870	0.899	**0.961**	**0.978**

The semantic similarities in BP ontology from eight different methods were compared in terms of correlations with the similarities of other functional features. To measure semantic similarity, IEA annotations were excluded.

**Table 4:** CESSM results of semantic similarities in MF ontology with IEA annotations.

Similarity	Resnik	Lin	Jiang	simUI	simGIC	G-SESAME	simICNP	simICND
Sequence	0.668	0.606	0.546	0.592	**0.717**	0.398	0.674	**0.710**
Pfam	0.572	0.564	0.491	0.618	0.638	0.498	**0.660**	**0.673**
ECC	0.603	0.642	0.561	0.637	0.622	0.714	**0.752**	**0.744**
Resolution	0.958	0.571	0.241	**0.967**	0.956	0.334	0.931	**0.963**

The semantic similarities in MF ontology from eight different methods were compared in terms of correlations with the similarities of other functional features. To measure semantic similarity, IEA annotations were included.

**Table 5:** CESSM results of semantic similarities in MF ontology without IEA annotations.

Similarity	Resnik	Lin	Jiang	simUI	simGIC	G-SESAME	simICNP	simICND
Sequence	0.651	0.598	0.522	0.591	0.666	0.439	**0.679**	**0.708**
Pfam	0.522	0.515	0.450	0.550	0.582	0.416	**0.605**	**0.587**
ECC	0.484	0.516	0.519	0.578	**0.587**	0.564	**0.598**	0.567
Resolution	0.934	**0.938**	0.364	0.930	0.936	0.595	0.830	**0.982**

The semantic similarities in MF ontology from eight different methods were compared in terms of correlations with the similarities of other functional features. To measure semantic similarity, IEA annotations were excluded.

The results of semantic similarities measured in BP ontology with and without IEA annotations are shown in Table 2 and 3. Top two semantic similarity methods for each reference feature (sequence, Pfam, Enzyme Commission classification, or resolution) are shown in bold. When compared to sequence similarity, Resnik's method and simGIC have slightly higher correlations than simICNP and simICND. However, for the other features, the two proposed methods outperform the others. When we compare Table 2 and 3, the similarities measured including IEA annotations have higher correlations than those without IEA annotations over all semantic similarity methods and features.

The results of semantic similarities measured in MF ontology with and without IEA annotations are shown in Table 4 and 5. Among previous methods, simGIC has relatively high correlations. However, when all features are considered, simICNP and simICND clearly show better results than simGIC. Similar to the results in BP ontology, the similarities measured including IEA annotations have higher correlations than those without IEA annotations over almost all semantic similarity methods and features. When we compare the results between BP and MF ontologies, the semantic similarities in BP ontology have higher correlations with sequence similarities, whereas the semantic similarities in MF ontology have higher correlations with Pfam domain and Enzyme Commission class similarities. Overall, the two new semantic similarity measures by merging well-performing previous methods yielded sensible improvements in the CESSM test.

#### PPI test

It has been verified that semantic similarity is also a good predictor of PPIs. The rationale behind this is that interacting protein pairs are likely to be involved in similar biological processes or molecular functions, and therefore should present higher values of semantic similarity than non-interacting protein pairs. Thus, given a positive set *P *of interacting protein pairs and a negative set *N *of non-interacting protein pairs, semantic similarity measures can be compared in terms of their ability to divide interacting and non-interacting protein pairs.

We validated simICND and simICNP on two PPI data sets of *S. cerevisiae *with different characteristics. First, a small, high quality positive set *P*_1 _of 11,936 interactions has been extracted from Hint [40], a database of manually reviewed PPIs. For the second data set, instead, the larger and more complete I2D [41] network was used as a positive set *P*_2_. I2D is a collection of interactions derived from several databases, and currently counts 147k interactions. The negative sets *N*_1 _and *N*_2 _were built by randomly selecting *|P*_1_*| *and *|P*_2_*| *protein pairs, respectively, not present in the iRefIndex [42] data set. iRefIndex is an index of 303k known, experimental or predicted PPIs that appear in a number of primary interaction databases. If a pair of proteins is not listed in iRefIndex, they are unlikely to be interacting with each other. Thus, selecting the negative set as pairs not present in iRefIndex should guarantee a low rate of false negatives. Given a cut-off threshold *k*, a linear separator predicts as interacting (*I*) all the protein pairs in *P *and *N *with the semantic similarity scores above *k*, and labels all the other protein pairs as non-interacting (*nI*).

Two significant indicators to compare the performance of different semantic similarity measures are sensitivity (*|P ∩ I|/|P |*) and specificity (*|N ∩ nI|/|N |*). The former, also called a true-positive rate, is the fraction of protein pairs in *P *whose scores are above the threshold. The latter, also called a true-negative rate, measures the fraction of proteins pairs in *N *whose scores are below the threshold. A false-positive rate is then calculated by 1-specificity. The true-positive and false-positive rates at different cut-off thresholds are collected and incorporated into a receiver operating characteristics (ROC) curve which is frequently used to evaluate prediction performance on a broad range of cut-off thresholds.

**Figure 1:**
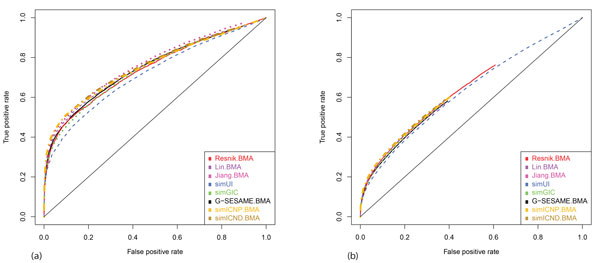
**PPI prediction test results of semantic similarities in BP ontology with and without IEA annotations**. Eight different methods were tested for PPI prediction by gradually decreasing the threshold of semantic similarity scores. The semantic similarities were measured in BP ontology (a) with IEA annotations and (b) without IEA annotations. In (a), simICND, simICNP and Jiang's method perform the best. In particular, simICND outperforms the others when the false-positive rate is less than 0.3.

**Figure 2:**
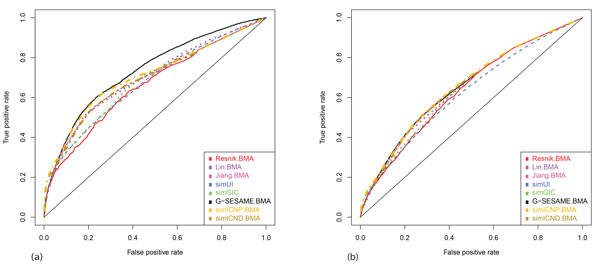
**PPI prediction test results of semantic similarities in MF ontology with and without IEA annotations**. Eight different methods were tested for PPI prediction by gradually decreasing the threshold of semantic similarity scores. The semantic similarities were measured in MF ontology (a) with IEA annotations and (b) without IEA annotations. In (a), simICNP and G-SESAME perform the best, and simICND, Jiang's method and Lin's method are included in the next group.

Figure 1 and 2 show the results of plotting ROC curves from eight different semantic similarity methods, which were also used in the CESSM test. The results of semantic similarities measured in BP ontology with and without IEA annotations are shown in Figure 1 (a) and 1(b). In Figure 1 (a), simICND, simICNP and Jiang's method showed the best performance in predicting PPIs. More precisely, simICNP has higher true-positive rates than Jiang's method when the false-positive rate is less than 0.2. Moreover, simICND has higher true-positive rates than Jiang's method when the false-positive rate is less than 0.3. Same to the CESSM results, the similarities measured including IEA annotations have better performance than those without IEA annotations over all semantic similarity methods. In Figure 1 (b), although all methods resulted in very similar plots, simICND and simICNP have slightly better performance than the others.

The results of semantic similarities measured in MF ontology with and without IEA annotations are shown in Figure 2 (a) and 2(b). In Figure 2 (a), simICND, simICNP and G-SESAME showed the best performance in predicting PPIs. G-SESAME works the best when the false-positive rate is greater than 0.4, whereas simICND and simICNP have better performance than G-SESAME when the false-positive rate is less than 0.1. Same to the test with BP ontology, the similarities measured including IEA annotations have better performance than those without IEA annotations over all semantic similarity methods. In Figure 2 (b), most methods resulted in similar plots of increasing true-positive rates. However, simICND and simICNP have slightly better performance when the false-positive rate is less than 0.1. Overall, the two new semantic similarity measures perform the best in predicting PPIs with strict threshold values.

### Performance evaluation of functional association mining

The performance of our functional association mining approach can be validated by comparing the outcome to functional modules. The genome-wide PPI data of several model organisms are publicly available from many open databases such as BioGRID [17], IntAct [43], MINT [44] and STRING [45]. In this performance test, we used the most recent version of the genome-wide PPI data set of *S. cerevisiae *from BioGRID, which includes 4,998 distinct proteins and 161,866 interactions. The first step is to weight PPIs. We used simICND since it has the best performance overall from the experiment shown in the previous section. Using a linear function, we transformed all simICND scores into the range between 0 and 1. Next, after selecting 1,000 proteins randomly for a query, we implemented our algorithm with each protein selected. In the information flow simulation, we assigned the initial score 1 to the query protein, and used 0.01 for the threshold to stop the flow on each linked path. As described earlier, we need additional parameter to select proteins and their links for a functional association network. (It will be called the minimum association threshold.) We made this threshold value variable, and examined how accuracy of our approach changes as the threshold changes.

Finally, 1,000 resultant functional association sub-networks were compared to functional modules. We used FunCat data from MIPS [46] as the functional modules of reference. Since this data set has been manually created, we assumed that it has the highest precision (also known as a positive predictive value). In other words, we assume that this data set rarely contains false positive proteins - the proteins that are included in the same functional module but do not perform the same functions. However, because this data set is not comprehensive and has not been updated recently, it is not guaranteed that it has the highest recall (also known as sensitivity). In other words, this data set might have many false negative proteins - the proteins that perform the same functions but are not included in the same functional module. In this performance test, we therefore measured precision only when the FunCat data set is used as gold-standard.

**Figure 3:**
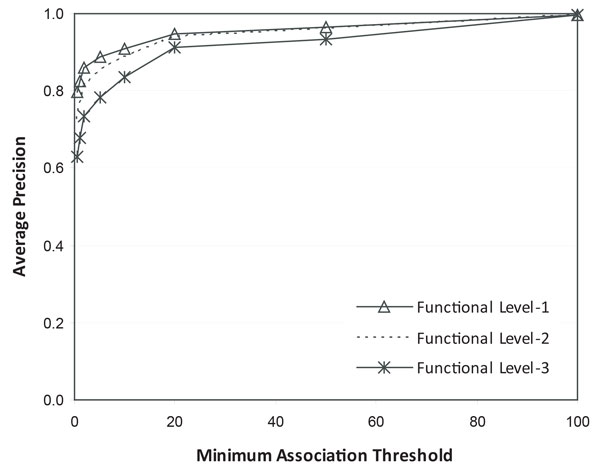
**Functional association mining results by M-Finder**. We weighted the yeast PPI network from BioGRID by simICND, and implemented the information flow-based algorithm using 1,000 random proteins in a query. For each output sub-network, we found the best match to functional modules in terms of precision (the ratio of the proteins in the output sub-network, which occur in the functional module). We finally calculated the average precision of all outputs and plotted it by altering the minimum association threshold. The reference functional modules on three different levels of functional hierarchy were obtained from MIPS. The modules with the most general descriptions are located on level-1, whereas those with the most specific functional descriptions are on level-3.

Figure 3 exhibits the average precision of detecting functionally associated proteins by 1,000 runs. The average precision increases as the minimum association threshold increases since the algorithm generates a smaller subgraph. High precision in a small subgraph means that most proteins in the subgraph strongly associated with the protein selected for a query. However, the precision increasing rate gradually declines when the threshold is greater than 20. And, when the threshold is greater than 60, subgraphs with a single node were generated in most runs.

The functional modules in FunCat are organized in a hierarchical tree structures. The first-level (i.e., top-level) modules indicate the most general descriptions of cellular functions whereas the bottom-level includes the most specific descriptions of cellular functions. We extracted the modules from the top three levels of the tree structure. The average precision of the results comparing to the functional modules on the three different levels is also shown in Figure 3. Although the three plots have a very similar increasing pattern, the highest precision can be achieved when the output subgraphs are compared to the largest modules with the most general functional descriptions because there is a higher chance that the output subgraphs include the proteins in the modules. Overall, the minimum association threshold between 15 and 20 is recommended to have high precision with relatively large sub-networks as output.

### Functional association mining software

We introduce the interactive web application software, called M-Finder, to analyze functional associations (functional linkage) from any protein of interest. This web-based tool is designed as an interactive system which enables a user to enter any protein in a query, choose a semantic similarity method, and specify the minimum association threshold as a parameter. Then the information flow algorithm, embedded in this tool, runs with the user inputs on the up-to-date genome-scale PPI network with edge weights, and the generated functional sub-network associated with the query protein is visualized.

The PPI data set is regularly updated with the most recent version from BioGRID [17]. The PPI weights are pre-computed by the semantic similarity methods and stored in our database. Since this large-scale PPI data set is likely to contain a large number of putative false positive interactions, we filter out the PPIs which have the semantic similarity score less than 0.1. This tool currently works for *S. cerevisiae *only, but it will be extended to *C. elegans*, *D. melanogaster *and *H. sapiens *in near future.

Since the information flow algorithm has a very quick response time (usually less than 10 seconds in our server) even on a large-scale network with complex connectivity, it is suitable for this interactive web-based tool. The threshold to halt the information flow was hard-coded as 0.01 in this tool because we observed this threshold is not sensitive to the result. However, the threshold to select the proteins and their links for a final functional association network should be a parameter that users can enter. The default of this threshold is 20, but it should decrease if a larger functional association network is needed.

**Figure 4:**
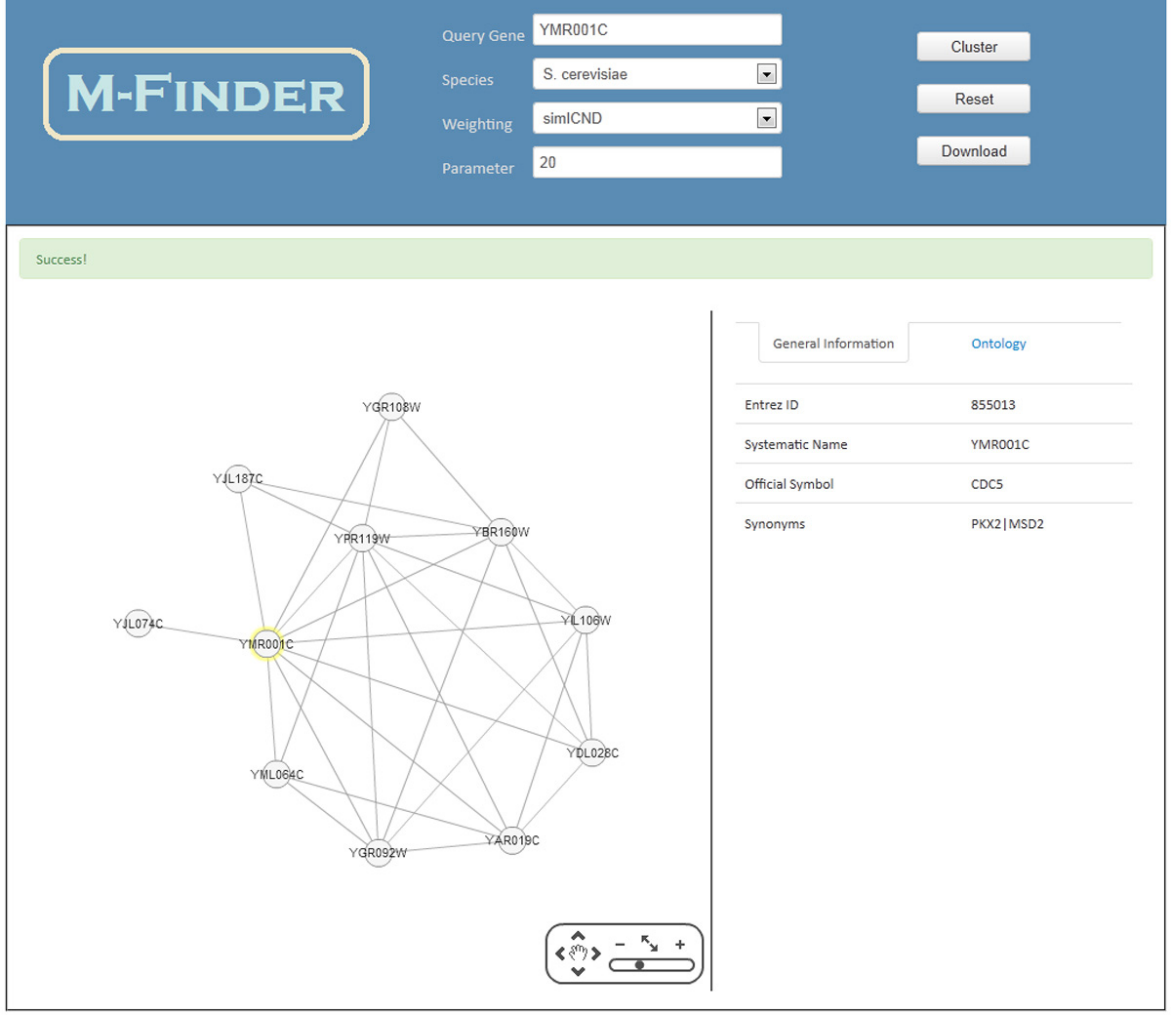
**The user interface of M-Finder**. On the top frame, a user can select the species and a weighting method (a semantic similarity measure), and enter a protein and a parameter value for the minimum association threshold in a query. The functional association network of the selected protein, which is represented as an undirected subgraph of the genome-wide PPI network, is visualized on the left frame. This tool also provides on the right frame the details of each interactor (protein) and each interaction in the visualized sub-network.

Cytoscape Web [47] is used for visualization of the functional association networks generated by our approach. This is a commonly used open-source platform to visualize networks. Its advantage is that it is easy to be adapted to our analysis and visualization by using the plug-in framework. Users can change flexibly the shape of resultant networks on Cytoscape Web and obtain different scales of the networks by increasing or decreasing the parameter value. Figure 4 shows the user interface of M-Finder. The functional linkage of the protein "YMR001C" is displayed when the weighting scheme of simICND and the parameterized threshold value of 20 are applied. It is known that "YMR001C" is involved in DNA synthesis, mitotic cell cycle and protein modification. The displayed functional association network shows that the protein in a query is mostly associated with the proteins having the functions of mitotic cell cycle and protein modification and forms a densely connected functional module with 7 proteins. In particular, "YMR001C" is strongly associated with the proteins for the G2/M transition of mitotic cell cycle, such as "YPR119W", "YJL187C", "YBR160W", "YGR108W" and "YJL074C".

M-Finder has further special features. Users can download the output functional association networks to any image files. Users can also search additional information of proteins and interactions on the output functional association network through hyperlinks. For example, ontological information related to the visualized proteins is provided.

## Conclusion

This article presented a novel computational method to analyze functional association patterns related to a user-specified protein in a query. This approach adopts the integration of interactome data and GO annotations, and the information flow algorithm reconstructs a functional association network linked to the query protein, which is a small subgraph of the genome-wide PPI network. As discussed in Introduction, a variety of graph clustering algorithms have been applied to detect functional modules from PPI networks. These graph clustering algorithms mostly search densely connected subgraphs assuming that proteins interact to perform a cellular function. However, listing all the clusters, i.e. the sets of proteins, in the genomic scale is not meaningful for genetic studies to characterize a specific gene or a specific function. The proposed approach is thus unique in that it investigates the patterns of potential functional linkage associated with a specific protein of interest. The introduced web application software to analyze and visualize functional association networks would be geared specifically to the needs of systematic and quantitative results in genetic studies.

This study has two significant contributions to current bioinformatics. First, biological data integration is increasingly demanding as an early stage of current data-intensive bioinformatics research. The automated high-throughput technologies have made rapid generation of large-scale data. However, as a downside, they decrease reliability of the data sets. It has been observed that the interactome data currently available in open databases include a large number of false positives, i.e., the spurious interactions which do not occur within a living cell. Although the high-throughput methods have produced interaction data over the entire genome scale, it is expected that there still exist an extremely large amount of false negatives across several model organisms, i.e., the actual interactions that have not been determined yet. The reliability of interaction data can be assessed by inspecting other resources which enable us to judge the feasibility of functional association between genes, such as gene expression profiles. We suggested, in this study, the integration of ontological data for filtering the interactome. We made a complete evaluation of recently proposed two integrative methods of semantic similarity, simICNP and simICND. The CESSM test and PPI test results demonstrated that the proposed approaches outperform the previous methods in terms of measuring functional closeness of two proteins. Our ontological data integration model would provide an effective framework for curation of genome, transcriptome, proteome and interactome data.

Second, efficiency and scalability are key issues on the large-scale, complex interactome data mining. A single protein influences multiple phenotypes in different environmental conditions, known as the pleiotropic effect. When separating the conditions is disregarded, interaction networks are typically structured by complex connectivity. Moreover, the scale of the interactome data increases remarkably for higher-level organisms in evolution. The proposed approach is formulated based off a data-mining technique which is implemented efficiently on large-scale networks with complex connectivity. Our heuristic model enables us to search functional associations very efficiently by simulating random walks. This efficient and scalable approach would be generalized to any integrative analysis of complex systems. This would also be the best fit to be embedded into a web application tool introduced in this article. For further improvement of the tool MFinder in terms of efficiency and accuracy, we can explore the inherent topological properties of the genome-wide PPI networks. It might be feasible to predict and suggest the best parameter value for a specific species by analyzing the properties.

## Competing interests

The authors declare that they have no competing interests.

## Authors' contributions

YRC coordinated the project, developed the methods, and drafted the manuscript. MM implemented semantic similarity evaluation and wrote the manuscript partially. YL developed application software, M-Finder. NK collected and curated PPI data. PHG designed semantic similarity evaluation. All authors revised and confirmed the manuscript.

## Declarations

The publication costs for this article were funded by the corresponding author.

This article has been published as part of *Proteome Science *Volume 11 Supplement 1, 2013: Selected articles from the IEEE International Conference on Bioinformatics and Biomedicine 2012: Proteome Science. The full contents of the supplement are available online at http://www.proteomesci.com/supplements/11/S1.
